# Supplementary material for: Solid-State Dewetting as a Driving Force for Structural Transformation and Magnetization Reversal Mechanism in FePd Thin Films
Source: Materials (Basel). 2022 Dec 22;16(1):92. doi: 10.3390/ma16010092 (PMC9821688; doi:10.3390/ma16010092)
Supplement: Supplementary file 1 [file materials-16-00092-s001.zip › materials-2086765-supplementary.pdf]

Supplementary Materials:

Solid state dewetting as a driving force for structural transformation and magnetization reversal mechanism in FePd thin film

Supplementary Materials S1. **XRD analysis**

Table S1 W dokumencie nie ma tekstu o podanym stylu.. Parameters extracted from XRD patterns showing lattice constant and coherence length for A1 and $L1_0$ phases after different times of annealing. Values in parentheses represent measurement uncertainties.

Time of annealing (min)	Percentage of $L1_0$ phase (%)	Cell parameters (Å) ±0.001			L_{coh} (nm)	
		a_{A1}	a_{L10}	c_{L10}	A1	$L1_0$
0	33.7	3.811	3.854	3.674	16.5(5)	11.9(4)
4	35.3	3.809	3.856	3.679	20.1(7)	10.4(2)
15	26.2	3.809	3.851	3.686	24.6(10)	12.0(6)
30	54.5	3.808	3.866	3.686	23.6(5)	11.5(8)
60	60.7	3.814	3.869	3.670	27.8(13)	18.7(6)
300	66.1	3.814	3.872	3.670	30.5(16)	22.1(11)

Supplementary Materials S2. **SEM**

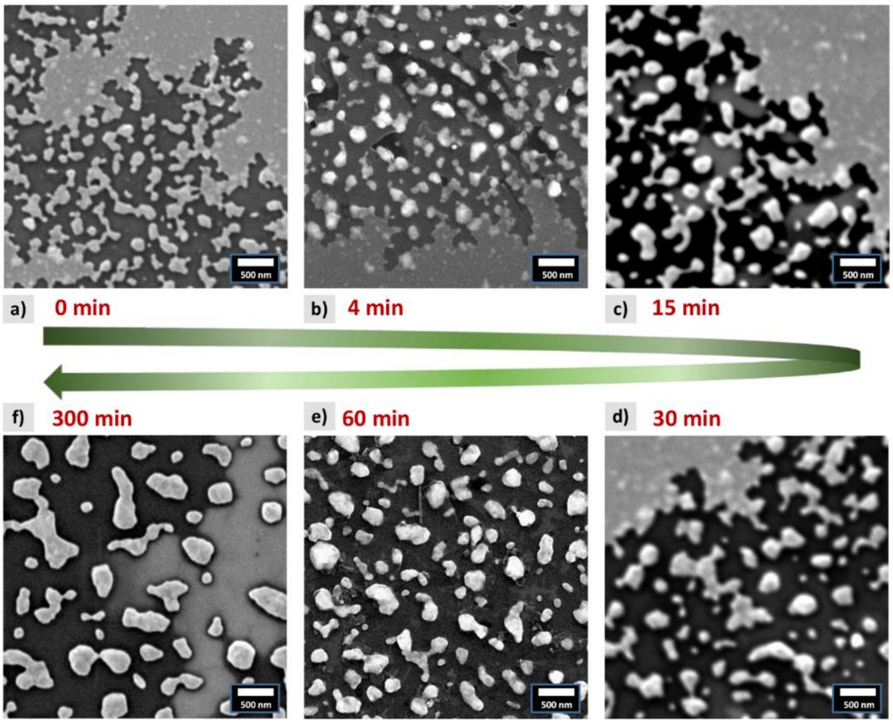

Figure S1. *SEM* images of FePd alloy annealed at 600^0C for different times.

Supplementary Materials S3. Switching field distribution fitting procedure.

The switching field distribution dependencies were tested for a different kind of models shown in Figure S2**.** Comparison between different fitting approaches of switching field distri. The asymmetric (red line) and symmetric (blue line) Lorentz distribution were used together with a composition of two independent maxima (green lines). The symmetric function gives less accurate results since it cannot reproduce stretched shape of the curve in direction of smaller (to the absolute value) magnetic fields. The model using two peaks gives relatively good fits but it is difficult to obtain systematic changes of all parameters for a series of all samples with the magnetic field applied between in-plane and out-of-plane directions (in total 60 independent measurements). A large number of freedom degrees and strong correlations between parameters resulted in random changes of parameters. Therefore, for all cases, an asymmetric Lorentz function was used.

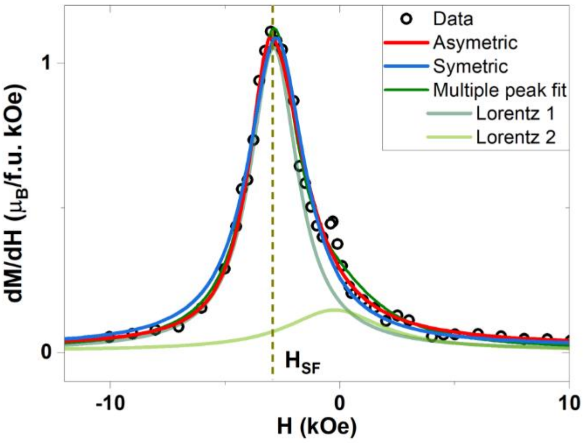

Figure S2. Comparison between different fitting approaches of switching field distribution.

Supplementary Materials S4. Models of magnetization reversal mechanism.

The magnetization reversal can be realized by rotation of magnetization of magnetic clusters or grains/particles, and by nucleation and movement of the magnetic domain walls. The rotation can be coherent (case of the Stoner-Wohlfarth model [1]), or incoherent, for example by curling [2], while the angular dependence of the reversal mechanism by domain wall motion is most often described with the Kondorsky model [3]. In some cases, a combination of two models is used. One approach is so-called the modified Kondorsky model for which a weighted sum of coherent rotation by Stoner-Wohlfarth and Kondorsky domain wall motion is proposed [4]. In this model, there is a critical angle of magnetization reversal for which a crossover between Kondorsky dominated model or coherent rotation model is realized. An alternative approach was proposed by Suponev et al. for ferromagnets with multidomain structures, the so-called M-type or M-shaped model. This type of dependence was already observed in alloys and thin films [5-7]. The comparison of Stoner-Wohlfarth coherent rotation (CR), Kondorsky domain wall motion (DWM) models and their two modifications, i.e. modified Kondorsky and M-type models are presented in Figure S3 and Equation S1.

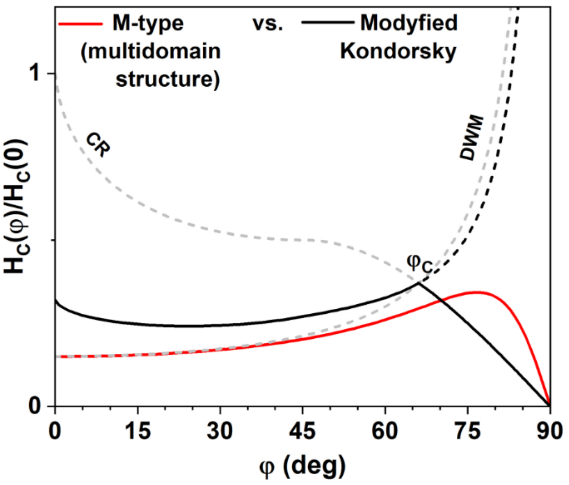

Figure S3. Comparison between angular dependences of coercivity field for modified Kondorsky (black line) and M-type magnetization rotation in multidomain ferromagnet models (red line).

Equation S1. Models of reversal magnetization mechanism:

- Stoner-Wohlfarth model of coherent rotation (CR):

$$h_C^{CR}(\varphi) = \begin{cases} \left| h_0^{CR}/(cos^{\frac{2}{3}}\varphi + sin^{\frac{2}{3}}\varphi)^{\frac{3}{2}} \right| & ,0 \leq \phi \leq \frac{\pi}{4} \\ 2\left| h_0^{CR}/(cos^{\frac{2}{3}}\frac{\pi}{4} + sin^{\frac{2}{3}}\frac{\pi}{4})^{\frac{3}{2}} \right| - & ,\frac{\pi}{4} \leq \phi \leq \frac{\pi}{4} \\ \left| h_0^{CR}/(cos^{\frac{2}{3}}\varphi + sin^{\frac{2}{3}}\varphi)^{\frac{3}{2}}) \right| & \end{cases}$$

- Kondorsky model of domain wall movement (DWM):

$$h_C^{DWM}(\varphi) = h_0^{DWM}/\cos\varphi$$

- Modified Kondorsky model:

$$h_C^{modK}(\varphi) = h_{eff} + \begin{cases} (1-r)h_C^{CR}(\varphi) + rh_C^{DWM}(\varphi), h_C^{modK}(\varphi) \leq h_C^{CR}(\varphi) \\ h_C^{CR}(\varphi) \qquad ,h_C^{modK}(\varphi) > h_C^{CR}(\varphi) \end{cases}$$

- Magnetization rotation in a multidomain ferromagnet (M-type model):

$$h_C^{M-type}(\varphi) = h_{eff} + \frac{h_0^{M-type}\cos\varphi}{Nsin^2(\varphi) + cos^2\varphi},$$

$$N = \frac{N_{easy-axis}}{N_{hard-axis} + N_A}$$

where the parameters h_0 are connected with the anisotropy energy necessary to rotate the magnetization vector of a magnetic particle at an angle $\varphi = 0^0$ (for CR model - h_0^{CR}) or to nucleate a reversed domain wall (in the case of DWM model - h_0^{DWM} or M-type model - h_0^{M-type}). In the case of the M-type model, the shape of the curve is strongly influenced by demagnetizing factors $N_{easy-axis}$, $N_{hard-axis}$ for the easy and hard direction of magnetization rotation, as well as a demagnetizing factor N_A arising from (mean) magnetocrystalline anisotropy.

During the fitting procedure the additional effective field (h_{eff}) parameter is used signifying a mean and angular independent constant field that arises for example from the magnetostatic field. This type of parameter was applied in a wide range of materials, i.e. hard granular magnets[8,9], arrays of nanowires [10], and nanoparticles [11].

Table S2a and S2b show parameters for the modified Kondorsky model and M-type model of a multidomain structure, respectively (see equation S1). In the case of the M-type model, additionally an angle φ_C^{M-type} is included indicating the point for which the fitted function reaches maximum. The modified Kondorsky model was fitted with ratio parameter r from equal to 100% corresponding to the situation for angles from 0 degrees up to the critical angle φ_C^{modK} only a domain wall motion mechanism is present, while above φ_C^{modK} only mechanism of magnetization rotation occurs. Therefore a φ_C^{modK} angle is a cut-off angle between the two mechanisms. Change of the parameter r results in an increase in the uncertainty of fitted parameters while the value of r did not drop below 95%.

Table S2a. Fitting parameters of reversal mechanism for modified Kondorsky model.

Time of annealing (min)	$H_0^{CR}/H_{SF}(0)$	H_0^{DWM} $/H_{SF}(0)$	H_{eff}^{modK} $/H_{SF}(0)$	φ_C^{modK} (deg.)	H_0^{DWM} $/H_0^{CR}$
0	13.8(24)	0.61(6)	0.39(6)	78.0	0.04(1)
4	5.80(57)	0.69(10)	0.32(9)	69.0	0.12(3)
15	2.94(24)	0.46(5)	0.54(4)	65.5	0.16(2)
30	3.78(62)	0.57(5)	0.44(5)	66.0	0.15(4)
60	1.33(12)	0.49(4)	0.48(4)	41.5	0.37(6)
300	1.10(12)	0.43(4)	0.58(5)	40.5	0.39(8)

Table S2b. Fitting parameters of reversal mechanism for M-type model of a multidomain structure.

Time of annealing (min)	H_0^{M-type} $/H_{SF}(0)$	N	H_{eff}^{M-type} $/H_{SF}(0)$	φ_C^{M-type} (deg.)
0	0.72(8)	0.02(1)	0.27(9)	81.5
4	0.83(19)	0.05(2)	0.18(11)	76.8
15	0.66(13)	0.09(3)	0.34(13)	71.5
30	0.80(13)	0.09(2)	0.23(13)	72.0
60	0.60(6)	0.28(3)	0.39(6)	51.5
300	0.56(6)	0.27(3)	0.46(6)	53.0

1. Stoner, E. C;. and Wohlfarth, E. P. A mechanism of magnetic hysteresis in heterogeneous alloys. *Philos. Trans. R. Soc. London. Ser. A, Math. Phys. Sci.* **1948**, 240, 599-642, doi:10.1098/rsta.1948.0007.
2. Aharoni, A. Angular dependence of nucleation by curling in a prolate spheroid. *J. Appl. Phys.* **1997**, 82, 1281-1287, doi:10.1063/1.365899.
3. Kondorsky, E. On hysteresis in ferromagnetics. *J. Phys.* **1940**, vol. 2, pp. 161.
4. Ratnam, D. V.; and Buessem, W. R. Angular Variation of Coercive Force in Barium Ferrite. *J. Appl. Phys.* **1972**, 43, 1291-1293, doi:10.1063/1.1661260.
5. Suponev, N. P.; Grechishkin, R. M.; Lyakhova, M. B.; and Pushkar, Y. E. Angular dependence of coercive field in (Sm,Zr) (Co,Cu,Fe) alloys. *J. Magn. Magn. Mater.* **1996**, 157–158, 376-377, doi:10.1016/0304-8853(95)00992-2.
6. Mathews, M.; Houwman, E. P.; Boschker, H.; Rijnders, G.; and Blank, D. H. A. Magnetization reversal mechanism in La0.67Sr0.33MnO3thin films on NdGaO3substrates. *J. Appl. Phys.* **2010**, 107, 0-5, doi:10.1063/1.3273409.
7. Han, X.M.; Ma, J.H.; Wang, Z.; Yao, Y.L.; Zuo, Y.L.; Xi, L.; Xue, D.S. Tunable in-plane uniaxial anisotropy and the magnetization reversal mechanism of patterned high-frequency soft magnetic FeTa strips. *J. Phys. D. Appl. Phys.* **2013**, *46*, 485004, doi:10.1088/0022-3727/46/48/485004.
8. Kronmüller, H.; Durst, K.-D.; Sagawa, M. Analysis of the magnetic hardening mechanism in RE-FeB permanent magnets. *J. Magn. Magn. Mater.* **1988**, *74*, 291–302, doi:10.1016/0304-8853(88)90202-8.

9. Givord, D.; Rossignol, M. F.; Taylor, D. W. Coercivity mechanisms in hard magnetic materials. *J. Phys. IV* **1992**, 02, C3-95-C3-104, doi:10.1051/jp4:1992314.
10. Viqueira, M.S.; Bajales, N.; Urreta, S.E.; Bercoff, P.G. Magnetization mechanisms in ordered arrays of polycrystalline Fe 100−x Co x nanowires. *J. Appl. Phys.* **2015**, *117*, 204302, doi:10.1063/1.4921701.
11. Komogortsev, S.V.; Fel'k, V.A.; Li, O.A. The magnetic dipole-dipole interaction effect on the magnetic hysteresis at zero temperature in nanoparticles randomly dispersed within a plane. *J. Magn. Magn. Mater.* **2019**, *473*, 410–415, doi:10.1016/j.jmmm.2018.10.091.
